# Supplementary material for: HIVprotI: an integrated web based platform for prediction and design of HIV proteins inhibitors
Source: J Cheminform. 2018 Mar 9;10:12. doi: 10.1186/s13321-018-0266-y (PMC5845081; doi:10.1186/s13321-018-0266-y)
Supplement: Supplementary file 2 — Additional file 2. Source code of HIVProtI web server. [file 13321_2018_266_MOESM2_ESM.zip › HIVprotI_Source-code/hivprotI/weka-3-6-6/doc/weka/core/Attribute.html]

Attribute


---


|  |  |  |
| --- | --- | --- |
| **Overview** \| **Package** \| **Class** \| **Tree** \| **Deprecated** \| **Index** \| **Help** \| **Weka's home** | |  |
| **PREV CLASS**   **NEXT CLASS** | **FRAMES**    **NO FRAMES**     **All Classes** |  |
| SUMMARY: NESTED \| FIELD \| CONSTR \| METHOD | DETAIL: FIELD \| CONSTR \| METHOD |  |


---


## weka.core Class Attribute

```
java.lang.Object
  weka.core.Attribute
```

**All Implemented Interfaces:**: java.io.Serializable, Copyable, RevisionHandler

---

``` public class Attribute extends java.lang.Object implements Copyable, java.io.Serializable, RevisionHandler ```

Class for handling an attribute. Once an attribute has been created,
it can't be changed.

The following attribute types are supported:

- numeric:
- nominal:   
  This type of attribute represents a fixed set of nominal values.
- string:   
  This type of attribute represents a dynamically expanding set of
  nominal values. Usually used in text classification.
- date:   
  This type of attribute represents a date, internally represented as
  floating-point number storing the milliseconds since January 1,
  1970, 00:00:00 GMT. The string representation of the date must be
  ISO-8601 compliant, the default is `yyyy-MM-dd'T'HH:mm:ss`.
- relational:   
  This type of attribute can contain other attributes and is, e.g.,
  used for representing Multi-Instance data. (Multi-Instance data
  consists of a nominal attribute containing the bag-id, then a
  relational attribute with all the attributes of the bag, and
  finally the class attribute.)

Typical usage (code from the main() method of this class):

`...   
// Create numeric attributes "length" and "weight"   
Attribute length = new Attribute("length");   
Attribute weight = new Attribute("weight");   
  
// Create vector to hold nominal values "first", "second", "third"   
FastVector my_nominal_values = new FastVector(3);   
my_nominal_values.addElement("first");   
my_nominal_values.addElement("second");   
my_nominal_values.addElement("third");   
  
// Create nominal attribute "position"   
Attribute position = new Attribute("position", my_nominal_values);  
...` 

**Version:**
:   $Revision: 5975 $

**Author:**
:   Eibe Frank (eibe@cs.waikato.ac.nz)

**See Also:**: Serialized Form

---

| **Field Summary** | |
| --- | --- |
| `static java.lang.String` | `ARFF_ATTRIBUTE`             The keyword used to denote the start of an arff attribute declaration |
| `static java.lang.String` | `ARFF_ATTRIBUTE_DATE`             The keyword used to denote a date attribute |
| `static java.lang.String` | `ARFF_ATTRIBUTE_INTEGER`             A keyword used to denote a numeric attribute |
| `static java.lang.String` | `ARFF_ATTRIBUTE_NUMERIC`             A keyword used to denote a numeric attribute |
| `static java.lang.String` | `ARFF_ATTRIBUTE_REAL`             A keyword used to denote a numeric attribute |
| `static java.lang.String` | `ARFF_ATTRIBUTE_RELATIONAL`             The keyword used to denote a relation-valued attribute |
| `static java.lang.String` | `ARFF_ATTRIBUTE_STRING`             The keyword used to denote a string attribute |
| `static java.lang.String` | `ARFF_END_SUBRELATION`             The keyword used to denote the end of the declaration of a subrelation |
| `static int` | `DATE`             Constant set for attributes with date values. |
| `static int` | `NOMINAL`             Constant set for nominal attributes. |
| `static int` | `NUMERIC`             Constant set for numeric attributes. |
| `static int` | `ORDERING_MODULO`             Constant set for modulo-ordered attributes. |
| `static int` | `ORDERING_ORDERED`             Constant set for ordered attributes. |
| `static int` | `ORDERING_SYMBOLIC`             Constant set for symbolic attributes. |
| `static int` | `RELATIONAL`             Constant set for relation-valued attributes. |
| `static int` | `STRING`             Constant set for attributes with string values. |


**Constructor Summary**

`Attribute(java.lang.String attributeName)`
  
          Constructor for a numeric attribute.

`Attribute(java.lang.String attributeName,
FastVector attributeValues)`
  
          Constructor for nominal attributes and string attributes.

`Attribute(java.lang.String attributeName,
FastVector attributeValues,
int index)`
  
          Constructor for nominal attributes and string attributes with
a particular index.

`Attribute(java.lang.String attributeName,
FastVector attributeValues,
ProtectedProperties metadata)`
  
          Constructor for nominal attributes and string attributes, where
metadata is supplied.

`Attribute(java.lang.String attributeName,
Instances header)`
  
          Constructor for relation-valued attributes.

`Attribute(java.lang.String attributeName,
Instances header,
int index)`
  
          Constructor for a relation-valued attribute with a particular index.

`Attribute(java.lang.String attributeName,
Instances header,
ProtectedProperties metadata)`
  
          Constructor for relation-valued attributes.

`Attribute(java.lang.String attributeName,
int index)`
  
          Constructor for a numeric attribute with a particular index.

`Attribute(java.lang.String attributeName,
ProtectedProperties metadata)`
  
          Constructor for a numeric attribute, where metadata is supplied.

`Attribute(java.lang.String attributeName,
java.lang.String dateFormat)`
  
          Constructor for a date attribute.

`Attribute(java.lang.String attributeName,
java.lang.String dateFormat,
int index)`
  
          Constructor for date attributes with a particular index.

`Attribute(java.lang.String attributeName,
java.lang.String dateFormat,
ProtectedProperties metadata)`
  
          Constructor for a date attribute, where metadata is supplied.


| **Method Summary** | |
| --- | --- |
| `int` | `addRelation(Instances value)`             Adds a relation to a relation-valued attribute. |
| `int` | `addStringValue(Attribute src, int index)`             Adds a string value to the list of valid strings for attributes of type STRING and returns the index of the string. |
| `int` | `addStringValue(java.lang.String value)`             Adds a string value to the list of valid strings for attributes of type STRING and returns the index of the string. |
| `java.lang.Object` | `copy()`             Produces a shallow copy of this attribute. |
| `Attribute` | `copy(java.lang.String newName)`             Produces a shallow copy of this attribute with a new name. |
| `java.util.Enumeration` | `enumerateValues()`             Returns an enumeration of all the attribute's values if the attribute is nominal, string, or relation-valued, null otherwise. |
| `boolean` | `equals(java.lang.Object other)`             Tests if given attribute is equal to this attribute. |
| `java.lang.String` | `formatDate(double date)`             Returns the given amount of milliseconds formatted according to the current Date format. |
| `java.lang.String` | `getDateFormat()`             Returns the Date format pattern in case this attribute is of type DATE, otherwise an empty string. |
| `double` | `getLowerNumericBound()`             Returns the lower bound of a numeric attribute. |
| `ProtectedProperties` | `getMetadata()`             Returns the properties supplied for this attribute. |
| `java.lang.String` | `getRevision()`             Returns the revision string. |
| `double` | `getUpperNumericBound()`             Returns the upper bound of a numeric attribute. |
| `boolean` | `hasZeropoint()`             Returns whether the attribute has a zeropoint and may be added meaningfully. |
| `int` | `index()`             Returns the index of this attribute. |
| `int` | `indexOfValue(java.lang.String value)`             Returns the index of a given attribute value. |
| `boolean` | `isAveragable()`             Returns whether the attribute can be averaged meaningfully. |
| `boolean` | `isDate()`             Tests if the attribute is a date type. |
| `boolean` | `isInRange(double value)`             Determines whether a value lies within the bounds of the attribute. |
| `boolean` | `isNominal()`             Test if the attribute is nominal. |
| `boolean` | `isNumeric()`             Tests if the attribute is numeric. |
| `boolean` | `isRegular()`             Returns whether the attribute values are equally spaced. |
| `boolean` | `isRelationValued()`             Tests if the attribute is relation valued. |
| `boolean` | `isString()`             Tests if the attribute is a string. |
| `boolean` | `lowerNumericBoundIsOpen()`             Returns whether the lower numeric bound of the attribute is open. |
| `static void` | `main(java.lang.String[] ops)`             Simple main method for testing this class. |
| `java.lang.String` | `name()`             Returns the attribute's name. |
| `int` | `numValues()`             Returns the number of attribute values. |
| `int` | `ordering()`             Returns the ordering of the attribute. |
| `double` | `parseDate(java.lang.String string)`             Parses the given String as Date, according to the current format and returns the corresponding amount of milliseconds. |
| `Instances` | `relation()`             Returns the header info for a relation-valued attribute, null if the attribute is not relation-valued. |
| `Instances` | `relation(int valIndex)`             Returns a value of a relation-valued attribute. |
| `void` | `setWeight(double value)`             Sets the new attribute's weight |
| `java.lang.String` | `toString()`             Returns a description of this attribute in ARFF format. |
| `int` | `type()`             Returns the attribute's type as an integer. |
| `boolean` | `upperNumericBoundIsOpen()`             Returns whether the upper numeric bound of the attribute is open. |
| `java.lang.String` | `value(int valIndex)`             Returns a value of a nominal or string attribute. |
| `double` | `weight()`             Returns the attribute's weight. |

**Methods inherited from class java.lang.Object**

`getClass, hashCode, notify, notifyAll, wait, wait, wait`

**Field Detail**

### NUMERIC

```
public static final int NUMERIC
```

:   Constant set for numeric attributes.

    **See Also:**: Constant Field Values

---


### NOMINAL

```
public static final int NOMINAL
```

:   Constant set for nominal attributes.

    **See Also:**: Constant Field Values

---


### STRING

```
public static final int STRING
```

:   Constant set for attributes with string values.

    **See Also:**: Constant Field Values

---


### DATE

```
public static final int DATE
```

:   Constant set for attributes with date values.

    **See Also:**: Constant Field Values

---


### RELATIONAL

```
public static final int RELATIONAL
```

:   Constant set for relation-valued attributes.

    **See Also:**: Constant Field Values

---


### ORDERING\_SYMBOLIC

```
public static final int ORDERING_SYMBOLIC
```

:   Constant set for symbolic attributes.

    **See Also:**: Constant Field Values

---


### ORDERING\_ORDERED

```
public static final int ORDERING_ORDERED
```

:   Constant set for ordered attributes.

    **See Also:**: Constant Field Values

---


### ORDERING\_MODULO

```
public static final int ORDERING_MODULO
```

:   Constant set for modulo-ordered attributes.

    **See Also:**: Constant Field Values

---


### ARFF\_ATTRIBUTE

```
public static final java.lang.String ARFF_ATTRIBUTE
```

:   The keyword used to denote the start of an arff attribute declaration

    **See Also:**: Constant Field Values

---


### ARFF\_ATTRIBUTE\_INTEGER

```
public static final java.lang.String ARFF_ATTRIBUTE_INTEGER
```

:   A keyword used to denote a numeric attribute

    **See Also:**: Constant Field Values

---


### ARFF\_ATTRIBUTE\_REAL

```
public static final java.lang.String ARFF_ATTRIBUTE_REAL
```

:   A keyword used to denote a numeric attribute

    **See Also:**: Constant Field Values

---


### ARFF\_ATTRIBUTE\_NUMERIC

```
public static final java.lang.String ARFF_ATTRIBUTE_NUMERIC
```

:   A keyword used to denote a numeric attribute

    **See Also:**: Constant Field Values

---


### ARFF\_ATTRIBUTE\_STRING

```
public static final java.lang.String ARFF_ATTRIBUTE_STRING
```

:   The keyword used to denote a string attribute

    **See Also:**: Constant Field Values

---


### ARFF\_ATTRIBUTE\_DATE

```
public static final java.lang.String ARFF_ATTRIBUTE_DATE
```

:   The keyword used to denote a date attribute

    **See Also:**: Constant Field Values

---


### ARFF\_ATTRIBUTE\_RELATIONAL

```
public static final java.lang.String ARFF_ATTRIBUTE_RELATIONAL
```

:   The keyword used to denote a relation-valued attribute

    **See Also:**: Constant Field Values

---


### ARFF\_END\_SUBRELATION

```
public static final java.lang.String ARFF_END_SUBRELATION
```

:   The keyword used to denote the end of the declaration of a subrelation

    **See Also:**: Constant Field Values


**Constructor Detail**

### Attribute

```
public Attribute(java.lang.String attributeName)
```

:   Constructor for a numeric attribute.

    **Parameters:**: `attributeName` - the name for the attribute

---


### Attribute

```
public Attribute(java.lang.String attributeName,
                 ProtectedProperties metadata)
```

:   Constructor for a numeric attribute, where metadata is supplied.

    **Parameters:**: `attributeName` - the name for the attribute: `metadata` - the attribute's properties

---


### Attribute

```
public Attribute(java.lang.String attributeName,
                 java.lang.String dateFormat)
```

:   Constructor for a date attribute.

    **Parameters:**: `attributeName` - the name for the attribute: `dateFormat` - a string suitable for use with SimpleDateFormatter for parsing dates.

---


### Attribute

```
public Attribute(java.lang.String attributeName,
                 java.lang.String dateFormat,
                 ProtectedProperties metadata)
```

:   Constructor for a date attribute, where metadata is supplied.

    **Parameters:**: `attributeName` - the name for the attribute: `dateFormat` - a string suitable for use with SimpleDateFormatter for parsing dates.: `metadata` - the attribute's properties

---


### Attribute

```
public Attribute(java.lang.String attributeName,
                 FastVector attributeValues)
```

:   Constructor for nominal attributes and string attributes.
    If a null vector of attribute values is passed to the method,
    the attribute is assumed to be a string.

    **Parameters:**: `attributeName` - the name for the attribute: `attributeValues` - a vector of strings denoting the attribute values. Null if the attribute is a string attribute.

---


### Attribute

```
public Attribute(java.lang.String attributeName,
                 FastVector attributeValues,
                 ProtectedProperties metadata)
```

:   Constructor for nominal attributes and string attributes, where
    metadata is supplied. If a null vector of attribute values is passed
    to the method, the attribute is assumed to be a string.

    **Parameters:**: `attributeName` - the name for the attribute: `attributeValues` - a vector of strings denoting the attribute values. Null if the attribute is a string attribute.: `metadata` - the attribute's properties

---


### Attribute

```
public Attribute(java.lang.String attributeName,
                 Instances header)
```

:   Constructor for relation-valued attributes.

    **Parameters:**: `attributeName` - the name for the attribute: `header` - an Instances object specifying the header of the relation.

---


### Attribute

```
public Attribute(java.lang.String attributeName,
                 Instances header,
                 ProtectedProperties metadata)
```

:   Constructor for relation-valued attributes.

    **Parameters:**: `attributeName` - the name for the attribute: `header` - an Instances object specifying the header of the relation.: `metadata` - the attribute's properties

---


### Attribute

```
public Attribute(java.lang.String attributeName,
                 int index)
```

:   Constructor for a numeric attribute with a particular index.

    **Parameters:**: `attributeName` - the name for the attribute: `index` - the attribute's index

---


### Attribute

```
public Attribute(java.lang.String attributeName,
                 java.lang.String dateFormat,
                 int index)
```

:   Constructor for date attributes with a particular index.

    **Parameters:**: `attributeName` - the name for the attribute: `dateFormat` - a string suitable for use with SimpleDateFormatter for parsing dates. Null for a default format string.: `index` - the attribute's index

---


### Attribute

```
public Attribute(java.lang.String attributeName,
                 FastVector attributeValues,
                 int index)
```

:   Constructor for nominal attributes and string attributes with
    a particular index.
    If a null vector of attribute values is passed to the method,
    the attribute is assumed to be a string.

    **Parameters:**: `attributeName` - the name for the attribute: `attributeValues` - a vector of strings denoting the attribute values. Null if the attribute is a string attribute.: `index` - the attribute's index

---


### Attribute

```
public Attribute(java.lang.String attributeName,
                 Instances header,
                 int index)
```

:   Constructor for a relation-valued attribute with a particular index.

    **Parameters:**: `attributeName` - the name for the attribute: `header` - the header information for this attribute: `index` - the attribute's index


**Method Detail**

### copy

```
public java.lang.Object copy()
```

:   Produces a shallow copy of this attribute.

    :   **Specified by:**: `copy` in interface `Copyable`
    :   **Returns:**: a copy of this attribute with the same index

---


### enumerateValues

```
public final java.util.Enumeration enumerateValues()
```

:   Returns an enumeration of all the attribute's values if the
    attribute is nominal, string, or relation-valued, null otherwise.

    :   **Returns:**: enumeration of all the attribute's values

---


### equals

```
public final boolean equals(java.lang.Object other)
```

:   Tests if given attribute is equal to this attribute.

    :   **Overrides:**: `equals` in class `java.lang.Object`
    :   **Parameters:**: `other` - the Object to be compared to this attribute **Returns:**: true if the given attribute is equal to this attribute

---


### index

```
public final int index()
```

:   Returns the index of this attribute.

    :   **Returns:**: the index of this attribute

---


### indexOfValue

```
public final int indexOfValue(java.lang.String value)
```

:   Returns the index of a given attribute value. (The index of
    the first occurence of this value.)

    :   **Parameters:**: `value` - the value for which the index is to be returned **Returns:**: the index of the given attribute value if attribute is nominal or a string, -1 if it is not or the value can't be found

---


### isNominal

```
public final boolean isNominal()
```

:   Test if the attribute is nominal.

    :   **Returns:**: true if the attribute is nominal

---


### isNumeric

```
public final boolean isNumeric()
```

:   Tests if the attribute is numeric.

    :   **Returns:**: true if the attribute is numeric

---


### isRelationValued

```
public final boolean isRelationValued()
```

:   Tests if the attribute is relation valued.

    :   **Returns:**: true if the attribute is relation valued

---


### isString

```
public final boolean isString()
```

:   Tests if the attribute is a string.

    :   **Returns:**: true if the attribute is a string

---


### isDate

```
public final boolean isDate()
```

:   Tests if the attribute is a date type.

    :   **Returns:**: true if the attribute is a date type

---


### name

```
public final java.lang.String name()
```

:   Returns the attribute's name.

    :   **Returns:**: the attribute's name as a string

---


### numValues

```
public final int numValues()
```

:   Returns the number of attribute values. Returns 0 for
    attributes that are not either nominal, string, or
    relation-valued.

    :   **Returns:**: the number of attribute values

---


### toString

```
public final java.lang.String toString()
```

:   Returns a description of this attribute in ARFF format. Quotes
    strings if they contain whitespace characters, or if they
    are a question mark.

    :   **Overrides:**: `toString` in class `java.lang.Object`
    :   **Returns:**: a description of this attribute as a string

---


### type

```
public final int type()
```

:   Returns the attribute's type as an integer.

    :   **Returns:**: the attribute's type.

---


### getDateFormat

```
public final java.lang.String getDateFormat()
```

:   Returns the Date format pattern in case this attribute is of type DATE,
    otherwise an empty string.

    :   **Returns:**: the date format pattern **See Also:**: `SimpleDateFormat`

---


### value

```
public final java.lang.String value(int valIndex)
```

:   Returns a value of a nominal or string attribute. Returns an
    empty string if the attribute is neither a string nor a nominal
    attribute.

    :   **Parameters:**: `valIndex` - the value's index **Returns:**: the attribute's value as a string

---


### relation

```
public final Instances relation()
```

:   Returns the header info for a relation-valued attribute,
    null if the attribute is not relation-valued.

    :   **Returns:**: the attribute's value as an Instances object

---


### relation

```
public final Instances relation(int valIndex)
```

:   Returns a value of a relation-valued attribute. Returns
    null if the attribute is not relation-valued.

    :   **Parameters:**: `valIndex` - the value's index **Returns:**: the attribute's value as an Instances object

---


### addStringValue

```
public int addStringValue(java.lang.String value)
```

:   Adds a string value to the list of valid strings for attributes
    of type STRING and returns the index of the string.

    :   **Parameters:**: `value` - The string value to add **Returns:**: the index assigned to the string, or -1 if the attribute is not of type Attribute.STRING

---


### addStringValue

```
public int addStringValue(Attribute src,
                          int index)
```

:   Adds a string value to the list of valid strings for attributes
    of type STRING and returns the index of the string. This method is
    more efficient than addStringValue(String) for long strings.

    :   **Parameters:**: `src` - The Attribute containing the string value to add.: `index` - the index of the string value in the source attribute. **Returns:**: the index assigned to the string, or -1 if the attribute is not of type Attribute.STRING

---


### addRelation

```
public int addRelation(Instances value)
```

:   Adds a relation to a relation-valued attribute.

    :   **Parameters:**: `value` - The value to add **Returns:**: the index assigned to the value, or -1 if the attribute is not of type Attribute.RELATIONAL

---


### copy

```
public final Attribute copy(java.lang.String newName)
```

:   Produces a shallow copy of this attribute with a new name.

    :   **Parameters:**: `newName` - the name of the new attribute **Returns:**: a copy of this attribute with the same index

---


### formatDate

```
public java.lang.String formatDate(double date)
```

:   Returns the given amount of milliseconds formatted according to the
    current Date format.

    :   **Parameters:**: `date` - the date, represented in milliseconds since January 1, 1970, 00:00:00 GMT, to return as string **Returns:**: the formatted date

---


### parseDate

```
public double parseDate(java.lang.String string)
                 throws java.text.ParseException
```

:   Parses the given String as Date, according to the current format and
    returns the corresponding amount of milliseconds.

    :   **Parameters:**: `string` - the date to parse **Returns:**: the date in milliseconds since January 1, 1970, 00:00:00 GMT **Throws:**: `java.text.ParseException` - if parsing fails

---


### getMetadata

```
public final ProtectedProperties getMetadata()
```

:   Returns the properties supplied for this attribute.

    :   **Returns:**: metadata for this attribute

---


### ordering

```
public final int ordering()
```

:   Returns the ordering of the attribute. One of the following:
    ORDERING\_SYMBOLIC - attribute values should be treated as symbols.
    ORDERING\_ORDERED - attribute values have a global ordering.
    ORDERING\_MODULO - attribute values have an ordering which wraps.

    :   **Returns:**: the ordering type of the attribute

---


### isRegular

```
public final boolean isRegular()
```

:   Returns whether the attribute values are equally spaced.

    :   **Returns:**: whether the attribute is regular or not

---


### isAveragable

```
public final boolean isAveragable()
```

:   Returns whether the attribute can be averaged meaningfully.

    :   **Returns:**: whether the attribute can be averaged or not

---


### hasZeropoint

```
public final boolean hasZeropoint()
```

:   Returns whether the attribute has a zeropoint and may be
    added meaningfully.

    :   **Returns:**: whether the attribute has a zeropoint or not

---


### weight

```
public final double weight()
```

:   Returns the attribute's weight.

    :   **Returns:**: the attribute's weight as a double

---


### setWeight

```
public void setWeight(double value)
```

:   Sets the new attribute's weight

    :   **Parameters:**: `value` - the new weight

---


### getLowerNumericBound

```
public final double getLowerNumericBound()
```

:   Returns the lower bound of a numeric attribute.

    :   **Returns:**: the lower bound of the specified numeric range

---


### lowerNumericBoundIsOpen

```
public final boolean lowerNumericBoundIsOpen()
```

:   Returns whether the lower numeric bound of the attribute is open.

    :   **Returns:**: whether the lower numeric bound is open or not (closed)

---


### getUpperNumericBound

```
public final double getUpperNumericBound()
```

:   Returns the upper bound of a numeric attribute.

    :   **Returns:**: the upper bound of the specified numeric range

---


### upperNumericBoundIsOpen

```
public final boolean upperNumericBoundIsOpen()
```

:   Returns whether the upper numeric bound of the attribute is open.

    :   **Returns:**: whether the upper numeric bound is open or not (closed)

---


### isInRange

```
public final boolean isInRange(double value)
```

:   Determines whether a value lies within the bounds of the attribute.

    :   **Parameters:**: `value` - the value to check **Returns:**: whether the value is in range

---


### getRevision

```
public java.lang.String getRevision()
```

:   Returns the revision string.

    :   **Specified by:**: `getRevision` in interface `RevisionHandler`
    :   **Returns:**: the revision

---


### main

```
public static void main(java.lang.String[] ops)
```

:   Simple main method for testing this class.

    :   **Parameters:**: `ops` - the commandline options


---


|  |  |  |
| --- | --- | --- |
| **Overview** \| **Package** \| **Class** \| **Tree** \| **Deprecated** \| **Index** \| **Help** \| **Weka's home** | |  |
| **PREV CLASS**   **NEXT CLASS** | **FRAMES**    **NO FRAMES**     **All Classes** |  |
| SUMMARY: NESTED \| FIELD \| CONSTR \| METHOD | DETAIL: FIELD \| CONSTR \| METHOD |  |


---
